# Prevalence of cardiovascular risk factors among Asian migrant workers in South Korea

**DOI:** 10.1371/journal.pone.0288375

**Published:** 2023-07-10

**Authors:** Pratibha Bhandari

**Affiliations:** School of Nursing and Midwifery, University of Technology Sydney, Sydney, New South Wales, Australia; Non-Communicable Diseases Research Center, Endocrinology and Metabolism Research Institute, Tehran University of Medical Sciences, ISLAMIC REPUBLIC OF IRAN

## Abstract

**Background:**

The burden of non-communicable diseases is rapidly increasing among young adults in middle- and low-income countries. Asian migrant workers continue to be a significant contributor to South Korea’s economy; however, their cardiovascular health is neglected. We explored the prevalence of cardiovascular risk factors among Asian migrant workers in South Korea.

**Methods:**

Cross-sectional survey, anthropometric measurements, blood pressure measurements, and biochemical tests including triglyceride, high-density lipoprotein, low-density lipoprotein, total cholesterol, fasting blood sugar, HbA1c, and C-reactive protein levels were conducted in 141 Asian migrant workers in South Korea.

**Results:**

The mean age of the participants was 31.3 (5.6) years. Of the participants, 14.8% were current smokers, and 47.5% consumed alcohol. The prevalence of overweight/obesity was 32.4%. The prevalence of hypertension and dyslipidemia were 51.2% and 64.6%, respectively. Of the participants, 98.5% had an increased waist circumference; elevated HbA1C and C-reactive protein was seen in 20.9% and 4.3%, respectively. The prevalence of metabolic syndrome was 5.5%. Clustering of two or more risk factors was seen in 45% of the participants. Factors associated with a high risk of cardiovascular diseases (clustering of two or more risk factors) were age (odds ratio 1.16, p < 0.01) and smoking (4.98, p < 0.05).

**Conclusion:**

The prevalence of cardiovascular risk factors was alarmingly high among Asian migrant workers employed in South Korea. Efforts to mitigate and eliminate those risk factors are urgently required.

## Introduction

The United Nations estimates that the majority of all international migrants worldwide originates from developing countries [1, 2]. South Korea is a popular destination for work-related migration by young, single Asians [3, 4]. Indeed, migrant workers are indispensable contributors to the economic growth of South Korea [4]. Due to changes in the sociodemographic structure and economy of South Korea such as the aging population and higher educational status, there is a need to fill a gap in the working population, especially in manual and labor-intensive areas. Since 2004, under the Employment Permit System (EPS), South Korea receives foreign workers from various Asian countries *e*.*g*., Sri Lanka, Bangladesh, Nepal, Thailand, Cambodia, Mongolia, Pakistan, China, Indonesia, Vietnam, and Myanmar. As of 2019, the total number of migrant workers per year was approximately 51,000–53,000 [5]. Migrant workers in South Korea are employed in various labor-intensive industries, such as agriculture, fisheries and livestock, manufacturing, and construction.

Labor migration occurs in most low-income developing countries [1], which is associated with challenges both in the country of origin and the host country, such as exploitation by the recruiting company in terms of illegal fees, resulting in debt, discrimination, and poor working and living conditions [6, 7]. Migrant workers typically engage in dangerous, difficult, and dirty jobs, which locals are reluctant to perform. This can lead to a variety of occupational health issues because of, for example, exposure to chemicals, occupational injuries, and chronic musculoskeletal diseases. Hence, occupational safety is the focus of research for migrant workers; many government resources and programs focus on this area [8]. By contrast, noncommunicable disease prevention is of lower priority.

Noncommunicable diseases account for > 70% of deaths worldwide [9]. South Asians have more cardiovascular risk factors and are at greater risk of cardiovascular diseases compared with Western or other Asian populations, such as Chinese and Japanese [10–13]. Increased visceral adiposity, dyslipidemia, and insulin resistance contributing to diabetes mellitus are common causes of cardiovascular diseases, in addition to genetic predisposition [11, 14, 15]. Because the burden of coronary artery disease is expected to increase, especially among middle- and low-income countries, identification of risk factors of coronary artery disease is important [10, 12]. The lipid profile -total cholesterol, high-density lipoprotein (HDL), low-density lipoprotein (LDL), triglycerides (TGs), levels of glucose, HbA1C, and C-reactive protein (CRP), and anthropometric measurements are predictive of cardiovascular risk in Asian populations [12, 14]. However, there have been no studies of cardiovascular health or its biomarkers among migrant workers in South Korea.

Psychological distress and depression are common among migrant workers [16, 17]. The prevalence of depression symptoms among migrant workers in South Korea is approximately 25–30% [17, 18]. Psychological distress can negatively affect cardiovascular health by accelerating atherosclerosis and stimulating release of stress hormones and cytokines [19] or indirectly by fostering negative health behaviors such as smoking, drinking alcohol, physical inactivity, and poor diet [20].

In South Korea, government resources and programs for migrant workers focus on occupational hazards and safety. Little or no attention is given to management and prevention of chronic disease. Despite much evidence of poor cardiovascular health, such as increased fatality rates due to cardiac arrest and suicide [3, 21] there is a dearth of research in this area. Most multicultural research in South Korea has focused on families with a Korean spouse [22] or white-collar workers [23]. Therefore, there is a paucity of research on the cardiovascular and mental health of migrant workers. Moreover, whereas the healthy immigration effect may decrease the risk of cardiovascular diseases in individuals with Korean spouses [24], this effect may not be pronounced in short-term residents. Hence, there is an urgent need for better means of screening and promoting the health of Asian migrant workers in South Korea.

This manuscript aims to provide baseline data by assessing the prevalence of cardiovascular risk factors among South Asian and Southeast Asian migrant workers in South Korea. The specific aims of the study are as follows:

### Aims of the study

Using a cross-sectional survey, physical assessments, and clinical laboratory data, we aimed to

1. Assess the prevalence of cardiovascular risk factors among South Asian and Southeast Asian migrant workers in South Korea.
2. Identify sociodemographic and behavioral factors associated with clustering of cardiovascular risk factors.

## Methods

### Study design, sample, and setting

A cross-sectional survey, anthropometric measurements, and biochemical tests were performed in four major cities densely populated by migrant workers. Participants were recruited through welfare and religious organizations that run welfare/support centers or religious services for migrant workers.

The inclusion criteria were (a) 18 years of age or older, (b) native of a South or Southeast Asian country, and (c) employed by a South Korean agriculture, livestock, machinery, production, or construction company. The exclusion criteria were (a) migrant workers who have transitioned to long-term or permanent resident status, (b) pregnancy, (c) any significant medical condition, and (d) taking medications that affect body composition (such as steroids).

**Fig 1:**
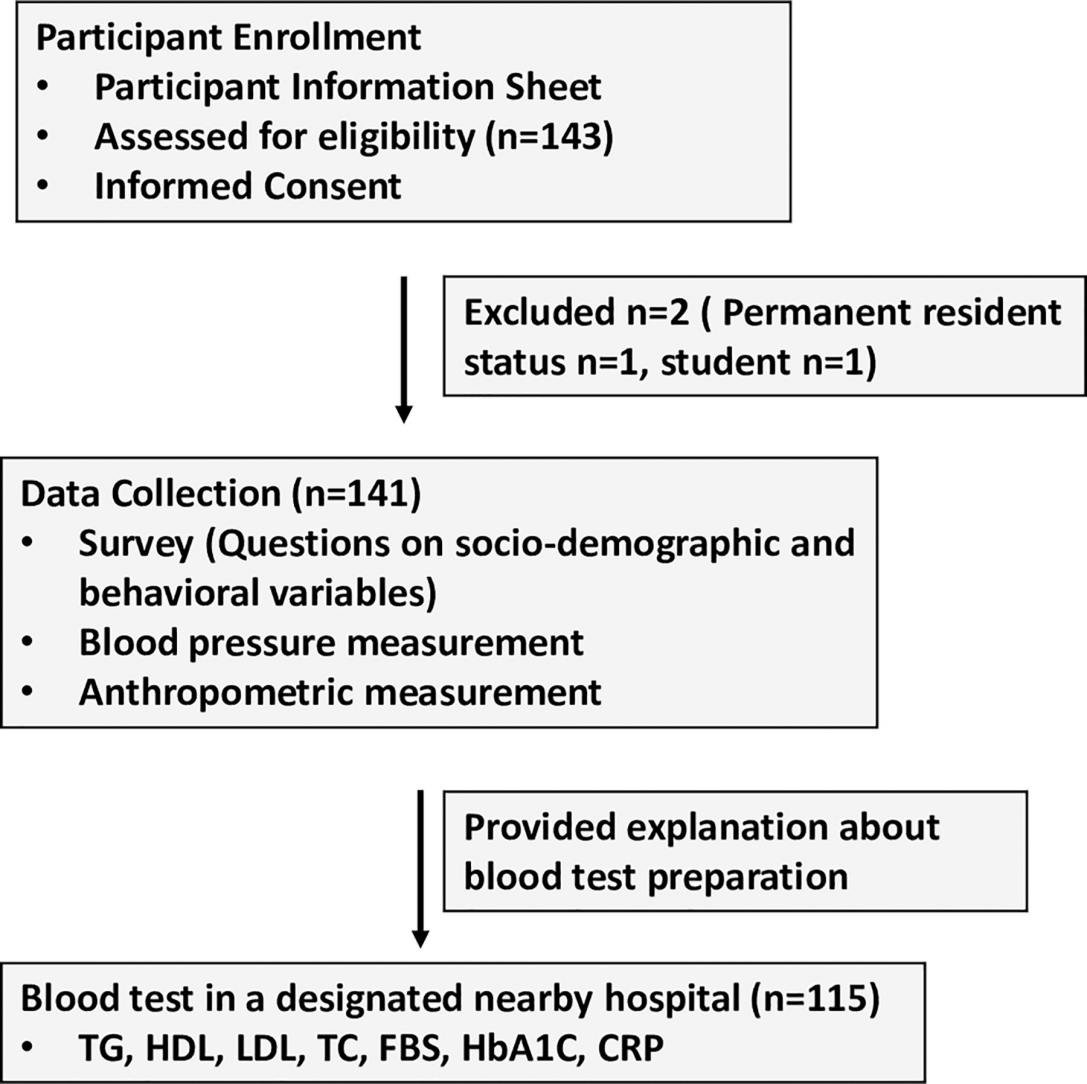
Flow diagram depicting the study process.

The aim and protocol of the study were explained to the participants. In addition, a participant information sheet explaining the research aims, process, option to opt out at any point of the study, contact details of the researcher was provided to all participants. All participants were reassured that not participating in the research would not affect any services that they get from the related welfare organizations. Individuals agreeing to participate were screened according to the inclusion criteria. Eligible participants were surveyed using the study questionnaire. Blood pressure was measured using an automatic digital blood pressure monitor after sitting for 10–15 minutes. Height, weight, and waist circumference were measured using standard methods. Finally, the participants were given instructions about overnight fasting and details of the nearby hospital where they were to provide a blood sample the next morning or within a week of the survey (Fig 1).

### Ethics approval and consent to participate

The research design was reviewed and approved by the institutional review board of the concerned university (IRB No.1041549-200707-SB-97). Written informed consent was obtained from all participants prior to data collection.

### Measurements

Sociodemographic and behavioral variables included age, gender, marital, education, and living statuses, job sector, monthly income, current smoking, alcohol, exercise frequency/week and type, and dietary intake. These variables were assessed using a questionnaire designed by the researcher. The measurements included anthropometric (height, weight, waist circumference) and blood pressure measurements. Biochemical variables (TG, HDL, LDL, total cholesterol, fasting blood sugar (FBS), HbA1c, and CRP levels), were measured in a designated nearby hospital.

### Data analysis

Statistical analysis was conducted using IBM SPSS 26.0 software. Data was checked for normality before progressing with the analysis using the Q-Q plot, Kolmogorov-Smirnov and Shapiro-Wilk tests. Simple descriptive statistics were calculated for all variables. Student’s t-test, chi-squared test, and Fisher’s exact test were used to compare the high- and low-risk groups. The prevalence of risk factors was calculated as a percentage with the 95% confidence interval. Factors associated with cardiovascular risk were estimated by logistic regression analysis. Statistical significance was set at *p* < 0.05. Clustering of cardiovascular risk factors was assessed as described below.

- *Metabolic syndrome*: The criteria for metabolic syndrome specified by American Heart Association (AHA) and the National Heart, Lung and Blood Institute (NHLBI) was used in this study. Metabolic syndrome was defined as the presence of three or more of the following criteria: waist circumference ≥ 40 inches for males and ≥ 35 inches in for females, triglyceride level ≥ 150 mg/dL, HDL level ≤ 40 mg/dL for males and ≤50 mg/dL for females, fasting blood glucose level ≥ 100 mg/dL, and blood pressure ≥ 130/85 mm Hg [25].
- *Dyslipidemia*: classified as the presence of at least one of the following: total cholesterol level > 200 mg/dL, triglyceride level > 150 mg/dL, LDL level > 130 mg/dL, and HDL level < 40 or < 50 mg/dL for males or females, respectively [26, 27].
- *HbA1c level* > 5.7%.
- *CRP level* > 0.5 mg/dL
- *Body mass index (BMI)* > 25
- *Current smoker*: those who reported smoking regularly or occasionally during the data collection period.

Cardiovascular risk factors were assessed based on the presence of metabolic syndrome, dyslipidemia, elevated HbA1C level (> 5.7%), elevated CRP level (> 0.5 mg/dL), BMI > 25, and current smoking. Participants were assigned to the high-risk group if they had two or more risk factors and to the low-risk group if they had one or zero risk factors.

## Results

The mean age of the participants was 31.3 (5.6) years. The mean duration of stay in South Korea was 4.03 (2.8) years. Most of the participants were males, married, and employed in the manufacturing sector; nearly half of the participants had a college degree.

Approximately 14.8% of the participants were current smokers, and almost half of the participants reported consuming alcohol on a regular or occasional basis. Less than half of the participants reported doing some form of exercise once or twice per week. The mean duration of each exercise session was 37.82 ± (34.6) minutes. The demographic characteristics are listed in Table 1.

**Table 1:** Demographic characteristics.

	N	Percentage	Mean (SD)
Age	141		31.31 (5.6)
Gender			
Male	122	87.8	
Female	17	12.2	
Country			
Nepal	96	68.6	
Cambodia	10	7.1	
Thailand	2	1.4	
Myanmar	8	5.7	
Bangladesh	24	17.1	
Marital Status			
Married	80	56.7	
Unmarried	61	43.3	
Highest level of education			
Primary	1	.7	
Secondary	20	14.2	
Higher secondary	56	39.7	
College or higher	64	45.4	
Living Status			
Alone	54	39.1	
With spouse	6	4.3	
Joint family	12	8.7	
Others (Company dormitory, With friends etc.)	66	47.8	
Religion			
Buddhist	37	26.4	
Christian	7	5	
Hindu	70	50	
Islam	22	15.7	
No religion	2	1.4	
Others	2	1.4	
Job Sector			
Agriculture	14	9.9	
Construction	7	5	
Manufacturing	117	83	
Others	3	2.1	
Perceived health			
Very good	5	3.5	
Good	74	52.5	
Fair	53	37.6	
Bad	9	6.4	
Current smoker			
Yes	21	14.8	
No	120	85.1	
Use of alcohol			
Yes	67	52.5	
No	74	47.5	
Exercise			
Yes	58	41.4	
No	38	27.1	
Occasionally	44	31.4	
Mean duration per exercise			37.82(34.61)
Number of times/weeks			3.26(2.08)
Consumption of fruits (Days/week)	140		3.43(2.19)
Fruits (Servings/day)	137		1.16 (.46)
Consumption of vegetables (Days/week)	140		3.52 (2.18)
Vegetables (Servings/day)	138		1.27 (.95)

### Anthropometric and biochemical variables

The prevalence of overweight (BMI 25–29.9) and obesity (BMI >30) were 30.2% and 2.2%, respectively. The prevalences of stage 1 and 2 hypertension were 38% and 21.2%, respectively. A very high percentage of the participants had an increased waist circumference (males > 101.6 cm, females > 88.9 cm). Dyslipidemia was seen in 64.6% of the participants. The prevalence of metabolic syndrome was 5.5%. The anthropometric and biochemical data are presented in Table 2.

**Table 2:** Anthropometric measurements and biochemical variables.

	Range	Mean (SD)
BMI	16.6–33.6	23.68(2.76)
Waist circumference (cm)	61–113	84.39(8.26)
Total cholesterol (mg/dl)	110–301	186.16(39.13)
HDL (mg/dl)	27–77	45.5(9.8)
LDL (mg/dl)	48–207	122.9(34.23)
Triglycerides (mg/dl)	35–426	116.85(71.86)
Fasting blood glucose (mg/dl)	71–131	95.3(14.7)
HbA1C (%)	4.5–8.3	5.4(.45)
CRP (mg/dl)	0.01–1.03	0.13(.14)

### Cardiovascular risk factors

Of the participants, 45% had a high risk of cardiovascular disease. Cardiovascular risk differed significantly according to age (t = −4.48, p < 0.01), gender, (Fisher’s exact test 5.20, p < 0.05), nationality (Fisher’s exact test 18.32, p < 0.001), marital status (Fisher’s exact test 16.22, p < 0.001), and smoking status (Fisher’s exact test 6.08, p < 0.01). The distribution of cardiovascular risk factors is presented in Table 3.

**Table 3:** Distribution of cardiovascular risk factors.

	N	Frequency	%
Waist circumference	137		
Increased		135	98.5
Normal		2	1.5
Blood pressure	137		
Normal (<120/<80 mmHg)		43	31.4
Elevated (120-129/<80mm Hg)		13	9.5
Stage 1 (130-139/80-89mm Hg)		52	38
Stage 2 (>140/>90 mmHg)		29	21.2
BMI	139		
Less than 18.5		6	4.3
18.5–24.9		88	63.3
25–29.9		41	29.5
30 or more		4	2.9
Triglycerides	115		
<150mg/dl		93	80.9
150-199mg/dl		9	7.8
>200 mg/dl		13	11.3
HDL	115		
<40 mg/dl		32	27.8
>40 mg/dl		83	72.2
LDL	115		
<100 mg/dl		24	20.9
100–129 mg/dl		48	41.7
130-159mg/dl		25	21.7
>160 mg/dl		18	15.7
Total cholesterol	115		
<200 mg/dl		76	66.1
200–239 mg/dl		28	24.3
>240 mg/dl		11	9.6
Fasting glucose	115		
<99 mg/dl		78	67.8
100–200 mg/dl		37	32.2
HbA1C			
<5.6%	115	91	79.1
5.7–6.4%		23	20
>6.5%		1	0.9
CRP	115		
<0.5mg/dl		110	95.7
>0.5mg/dl		5	4.3
Consumption of fruits (Days/week)	140	Mean (SD) 3.43(2.19)	
Fruits (Servings/day)	137	1.16 (.46)	
Consumption of vegetables (Days/week)	140	3.52 (2.18)	
Vegetables (Servings/day)	138	1.27 (.95)	

* (> 40 inches for males, >35 inches for females)

### Binary logistic regression analysis

Logistic regression was performed to identify factors associated with clustering of two or more risk factors. The independent variables in the model were age, gender, nationality, and marital, smoking, and drinking statuses. The full model containing all predictors was significant (χ^2^ = 32.9, p < 0.001) and explained 37.4% of the variance in cardiovascular disease risk. The significant predictors of high risk of cardiovascular disease were age (OR = 1.16, p < 0.01) and smoking (OR = 4.98, p < 0.05). Respondents who were older or who smoked were 1.16- or 4.98-fold, respectively, more likely to have a high risk of cardiovascular disease. These results are listed in Table 4.

**Table 4:** Logistic regression.

Variables	B	S.E.	Wald	df	*p*	Exp(B) OR	95% CI
Age	.153	.057	7.161	1	**.007**	**1.165**	1.04–1.30
Gender-female	-.785	.835	.884	1	.347	.456	.089–2.34
Nationality	-1.752	1.313	1.780	1	.182	.173	.013–2.27
Marital Status	-.631	.660	.914	1	.339	.532	.146–1.94
Current smoker: No	1.606	.702	5.240	1	**.022**	**4.984**	1.260–19.71
Use of alcohol: No	.036	.528	.005	1	.945	1.037	.369–2.91
Constant	-4.978	1.973	6.367	1	.012	.007	

## Discussion

Migrant workers are significant contributors to the host country’s economy but are vulnerable and disadvantaged. Health care for migrant workers has long focused on occupational health. In South Korea, the increasing numbers of sudden deaths and suicides among migrant workers have been highlighted in the media. However, there are no studies on cardiovascular disease risk factors. This is the first study using both survey and laboratory data to highlight the burden of non-communicable diseases among young Asian migrant workers in South Korea.

The most prevalent risk factor was dyslipidemia (64.6%), followed by overweight/obesity (32.4%) and an increased HbA1C level (20.9%). Clustering of two or more risk factors was seen in 45% of the participants. In a similar study conducted in a rural Nepalese population aged 40–80 years [28], dyslipidemia was reported in approximately 56% of the participants, overweight/obesity in 59.4%, and diabetes in 16.2%. South Asians have higher TG and lower HDL levels compared with Caucasians [10]; the pattern was slightly different in this study. The mean age of the participants was 31.3 years, indicating that the burden of cardiovascular disease is increasing at an alarming rate in the younger population. The average number of working hours per day of a migrant worker employed in small and medium businesses is 11.4 hours, plus overtime and weekend work, which hinders engaging in physical activity during leisure time. In addition, migrant workers are employed in labor-intensive areas, increasing their fatigue and risk of participating in negative health behaviors such as drinking alcohol or smoking. All employers hiring workers through the EPS are required to add them to the national health insurance program, which includes a biennial general health screening. Lipid profile, anthropometric, and blood pressure measurements are performed; however, other than obtaining the Korean report by mail, a physician recommendation cannot be obtained. Improvements in post-health checkup consultations may be beneficial in this group of adults.

Smoking was reported by 14.8% of the participants. This rate is slightly higher than that in Canada [29] and lower than that in Thailand [30] and South Asian migrants working in the United Arab Emirates (UAE) [31, 32]. In South Korea, the proportion of people (15 years or older) who smoke daily is 16.4%; this is slightly lower than the rate the host country [32].

Consumption of alcohol was reported by almost half of the participants (47.5%). This is higher than the rate among migrant workers from the UAE; similarly, in Nepal the prevalence of harmful use of alcohol was 10.7%. The higher percentage of alcohol consumption in this study could be due to the negative cultural effect of living in a country where harmful use of alcohol is a major public health issue among working adults [33].

The prevalence of overweight/obesity was lower in this study compared with those in migrant workers from the UAE [13, 31], Bangladesh [34], and Malaysia [35]. In South Asian migrants, low physical activity, increased abdominal obesity, dyslipidemia, and type 2 diabetes are risk factors for cardiovascular disease [11, 12]. Thus, a heathy lifestyle and means of preventing dyslipidemia and other risk factors must be prioritized in migrant workers in South Korea.

High blood pressure (stage 1 and 2) was seen in 59% of the participants, a higher rate than that in the STEPS survey in Nepal (24.8%) [36] and Vietnam (18.9%) [37]. The higher rates of risk factors in this study may explain the greater prevalence of high blood pressure. In addition, psychological stress, which is common in migrant workers, may have contributed to the prevalence of high blood pressure [6, 7, 38].

As reported previously [39], our results showed that fruit and vegetable intake was insufficient. Of the participants, 2.7% consumed five or more servings of fruits and vegetables/day [40]. The prevalence of metabolic syndrome was 5.5%, perhaps related to dietary practices such as consumption of refined carbohydrates, low protein intake, and inadequate intake of fruits/vegetables, as well as physical inactivity [41].

The logistic regression analysis revealed that age and smoking increased the odds of belonging to the high-risk group (two or more risk factors) by 1.16- and 4.98-fold, respectively. Cardiovascular risk increases with age [11, 42]. The EPS program accepts migrant workers aged 18–39 years. Urgent preventive measures tailored to South and Southeast Asian young adults are needed to decrease the burden on the healthcare system due to noncommunicable diseases. Awareness of the correlations between risk factors and non-communicable diseases is important. The findings highlight the need to relay information on and reinforce the negative health impacts of smoking; workplace policies to discourage smoking may also be useful.

The healthy migrant effect posits that migrants, despite being a socioeconomically disadvantaged group, are in better health than the host population [43, 44]. In this study, no such effect was observed; the prevalence of cardiovascular risk factors was higher than that in the general population of South Korea. The prevalences of hypertension, diabetes, and dyslipidemia are reported to be 23.44%, 8.33%, and 16.8%, respectively [42]. This could be because adults migrating for short-term blue-collar work may not be a healthy population; it may also indicate gaps in the migration-related health checkups conducted in the country of origin [45].

Most participants had more than two risk factors for cardiovascular disease, raising concern over the cardiometabolic disease burden among migrant workers in South Korea. A healthy lifestyle decreases the risk of cardiovascular diseases by 66% [46]; hence, implementation of interdisciplinary interventions targeting modifiable risk factors in collaboration with local employers/government is recommended to prevent cardiovascular events. Migrating for work is costly and involves leaving one’s family in their country or origin. Simple tailored interventions would significantly reduce the physical, emotional, and economic burden on the individual, family, host country, and country of origin.

This study had several limitations. The sample size was small. Much effort was expended to recruit representative participants from various areas; however, this was hampered by the coronavirus disease 2019 pandemic. Thus, the study population may not be representative of migrant workers in South Korea. In addition, as this was a cross-sectional study, casual inferences cannot be made. Future prospective studies are needed.

Although we attempted to adapt the WHO regulations (STEPS), not all measurements were conducted in accordance with WHO specifications. For example, only one blood-pressure reading was taken, after the participants had rested for approximately 15 minutes. However, the mean of three readings would have been more accurate. In addition, detailed information on physical activity (measured in MET) was not obtained and daily physical activity as a part of their work was not considered. It is recommended that future studies should be conducted with the inclusion of these variables.

## Conclusion

The study assessed the cardiovascular risk factors in South and Southeast Asian migrant workers in South Korea. The results provides epidemiological evidence that cardiovascular risk factors was alarmingly high among Asian migrant workers employed in South Korea. Most of the participants had more than two risk factors. Efforts to mitigate and eliminate these risk factors are needed. Increasing awareness of cardiovascular risk factors and its prevention through various outreach programs, developing screening and preventive programs targeted to migrant workers both at the home and destination countries are urgently required. Our findings will inform the formulation of policies and preventive measures to improve the cardiovascular health of migrant workers in South Korea.

## Supporting information

S1 Dataset (XLSX)
